# Neutralizing Antibodies and Cytokines in Breast Milk After Coronavirus Disease 2019 (COVID-19) mRNA Vaccination

**DOI:** 10.1097/AOG.0000000000004661

**Published:** 2021-11-30

**Authors:** Vignesh Narayanaswamy, Brian T. Pentecost, Corina N. Schoen, Dominique Alfandari, Sallie S. Schneider, Ryan Baker, Kathleen F. Arcaro

**Affiliations:** Department of Veterinary and Animal Sciences, University of Massachusetts, Amherst, and Maternal Fetal Medicine and Pioneer Valley Life Sciences Institute, Baystate Medical Center, Springfield, Massachusetts.

## Abstract

**OBJECTIVE:**

To evaluate immune responses to coronavirus disease 2019 (COVID-19) mRNA-based vaccines present in breast milk and transfer of the immune responses to breastfeeding infants.

**METHODS:**

We enrolled 30 lactating women who received mRNA-based COVID-19 vaccines from January through April 2021 in this cohort study. Women provided serial milk samples, including milk expressed before vaccination, across 2–3 weeks after the first dose, and across 3 weeks after the second dose. Women provided their blood, spotted on cards (dried blood spots), 19 days after the first dose and 21 days after the second dose. Stool samples from the breastfed infants were collected 21 days after mothers' second vaccination. Prepandemic samples of milk, dried blood spots, and infant stool were used as controls. Milk, dried blood spots, and infant stool were tested by enzyme-linked immunosorbent assay for receptor-binding domain (RBD)–specific immunoglobulin (Ig)A and IgG. Milk samples were tested for the presence of neutralizing antibodies against the spike and four variants of concern: D614G, Alpha (B.1.1.7), Beta (B.1.351), and Gamma (P.1). Levels of 10 cytokines were measured in milk samples.

**RESULTS:**

Milk from COVID-19-immunized women neutralized the spike and four variants of concern, primarily driven by anti-RBD IgG. The immune response in milk also included significant elevation of interferon-γ. The immune response to maternal vaccination was reflected in breastfed infants: anti-RBD IgG and anti-RBD IgA were detected in 33% and 30% of infant stool samples, respectively. Levels of anti-RBD antibodies in infant stool correlated with maternal vaccine side effects. Median antibody levels against RBD were below the positive cutoffs in prepandemic milk and infant stool samples.

**CONCLUSION:**

Humoral and cellular immune responses to mRNA-based COVID-19 vaccination are present in most women’s breast milk. The milk anti-RBD antibodies can neutralize severe acute respiratory syndrome coronavirus 2 (SARS-CoV-2) spike and variants of concern. Anti-RBD antibodies are transferred to breastfed infants, with the potential to confer passive immunity against SARS-CoV-2.

## Abstract 2

Humoral and cellular immune responses to mRNA-based coronavirus disease 2019 (COVID-19) vaccination are present in the breast milk of most women and confer passive immunity to the nursing infant.

Two coronavirus disease 2019 (COVID-19) mRNA-based vaccines, the BNT162b2 (Pfizer-BioNTech) and mRNA-1273 (Moderna), are approved for use in the United States.^1,2^ Clinical trials for the two mRNA vaccine candidates did not include breastfeeding women, but the Centers for Disease Control and Prevention, the American College of Obstetricians and Gynecologists, and the Academy of Breastfeeding Medicine recommend that breastfeeding women receive the vaccine.^3,4^ At the time of writing this article, given that COVID-19 vaccines are not yet approved for children younger than 5 years of age, passive immunity conferred to breastfeeding infants by vaccinated mothers, or to the developing fetus through the placenta of vaccinated pregnant women, are likely the only means of protection for infants from severe acute respiratory syndrome coronavirus 2 (SARS-CoV-2) infection.

New genetic variants of concern from the original SARS-CoV-2 sequence have emerged in the past several months. The D614G variant is associated with increased infectivity,^5^ the Alpha variant (B.1.1.7) is associated with enhanced transmissibility,6 and some reports suggest that the Beta and Gamma variants (B.1.351 and P.1) evade the natural immunity conferred by a prior SARS-CoV-2 infection.^7,8^

We identified 10 published studies on the immune response to an mRNA-based COVID-19 vaccine in breast milk of lactating women.^9–18^ None of these studies measured cellular responses to the COVID-19 vaccine in milk or the presence of anti–SARS-CoV-2–specific antibodies in breastfed infants. We assessed levels of anti–receptor-binding domain (RBD) immunoglobulin (Ig)A and IgG in serial milk samples and neutralizing capacity against the wildtype spike and four SARS-CoV-2 variants in prevaccination and postvaccination milk samples. We also assessed the vaccinated mothers’ milk for changes in levels of 10 cytokines and the infant stool for levels of anti-RBD IgA and IgG.

## METHODS

Study details were promoted on the website breastmilkresearch.org. Individuals from across the continental United States could enroll if they were lactating and scheduled to receive the vaccine or were recently vaccinated with either the mRNA-based BNT-162b2 (Pfizer-BioNTech) or mRNA-1273 (Moderna) A total of 30 women were enrolled in the study and signed a UMass Amherst Institutional Review Board–approved consent form, meeting the goal of recruiting a minimum of 10 women for each vaccine brand. To assess variables potentially associated with responses to vaccination, women were asked to complete questionnaires on demographics (eg, age and race), general health, lactation status, previous SARS-CoV-2 infection and related symptoms, type of COVID-19 vaccine received, and the timing of COVID-19 vaccination and related maternal and infant side effects. Information was logged in REDCap (Research Electronic Data Capture).

To establish baseline anti-RBD IgG and IgA levels, we included matched breast milk, dried blood spots, and infant stool samples. The prepandemic set of milk samples was established from 12 women who donated milk between January 2018 and September 2019 as part of a different Institutional Review Board–approved study at UMass Amherst. The prepandemic set of dried blood spot cards was established from eight reproductive-aged women who provided blood between January 2017 and August 2019 as part of a separate Institutional Review Board–approved study at Baystate Medical Center. Finally, the prepandemic set of stool samples was established from six infants, collected during August 2019 as part of a previous Institutional Review Board–approved study at UMass Amherst. Prepandemic milk, maternal dried blood spots, and infant stool samples were collected using the same methods as samples in the vaccine cohort.

Consented participants were sent kits with instructions for sample collection, storage, and return. Women were asked to provide serial bilateral breast milk samples at 13 (Pfizer) or 15 (Moderna) timepoints (approximately every 3 days) over 42 (Pfizer) or 48 (Moderna) days. The timepoints of milk donation varied for each woman (Appendix 1, available online at http://links.lww.com/AOG/C528). Participants were instructed to freeze their milk after expression at each timepoint until all samples were collected and were ready to be shipped to UMass Amherst. Milk samples on 2 consecutive days before receiving the first vaccine dose were requested from women. In addition to milk samples, women also were asked to provide a sample of their blood at two timepoints—19 days after receiving the first dose and 21 days after receiving the second dose. Women who consented were asked to collect blood samples on spot cards (dried blood spots) (Whatman FTA card, #WHAWB120205), which were left to dry at room temperature. Consenting women also provided their infants’ stool samples collected 21 days after the mothers received their second dose. Infant stool samples were collected in stool-collection tubes (Fisher Sci., Cat No. NC0705093) containing 8 mL of 95% ethanol. After all samples were collected, participants packaged the samples with ice packs in the kit provided and shipped them to UMass Amherst using an overnight express courier.

Equal volumes of bilateral milk samples were mixed to generate a combined sample. Briefly, 500 microliters of combined milk were centrifuged at 820 g for 8 minutes. The whey fraction was carefully transferred to a 48-well plate, and samples from the plate were used for the detection of SARS-CoV-2 RBD-specific immunoglobulins and cytokines and in the neutralization assay.

Discs (6 mm diameter) prepared from spot cards were transferred to a 24-well plate. Five hundred microliters of TBST (Tris-buffered saline with 0.05% Tween 20) were added to the dried blood spots discs, and the plate was incubated with gentle shaking overnight at 4°C. Samples of blood spot eluates were used for the detection of SARS-CoV-2 RBD-specific immunoglobulins.

Infant stool samples were received in stool-collection tubes containing 95% ethanol. The tube containing stool was vortexed for 20 minutes until a homogenous suspension was achieved. Aliquots of the stool samples were prepared and stored at −20°C. A single aliquot was retrieved at the time of analysis and centrifuged at 4,000 g for 20 minutes at 4°C. After centrifugation, the ethanol supernatant was aspirated, leaving behind the stool pellet, to which TBST was added. The tube was vortexed for 5 minutes, centrifuged at 4,000 g for 20 minutes at 4°C, and the TBST supernatant was used for the detection of total and SARS-CoV-2 RBD-specific immunoglobulins.

Levels of SARS-CoV-2 RBD-specific immunoglobulins were measured as previously described using enzyme-linked immunosorbent assays developed and validated at UMass Amherst.^19^ The RBD on the virus spike protein is involved in docking of virus to cells, elicits a clear immune response, and is the assayed target in most commercial screening tests. For detection of total immunoglobulins, 96-well plates were coated with anti-IgA (α-chain specific) or anti-IgG (H+L specific) capture antibodies at 1 microgram/mL. Subsequent steps followed the protocol for detection of RBD-specific immunoglobulins.^19^

The neutralization assay was performed using a V-PLEX SARS-CoV-2 Panel 6 multiplex assay by Mesoscale Discovery (K15436U). The assay quantitatively measures antibodies in the sample that can inhibit the interaction of spike and its variants with ACE2. Each plate included an 8-point standard curve. We are reporting results for wildtype spike (referred to as spike) and four spike variants: D614G, B.1.1.7 (Alpha), B.1.351 (Beta), and P.1 (Gamma). All samples and standards were run in technical duplicates. We measured neutralizing ability in milk from 28 of the 30 women who provided samples both before the first dose and after the second dose. For the second time point, the milk sample with the highest level of anti-RBD IgG was selected for each woman—typically the final, penultimate, or antepenultimate sample collected (Appendix 1, http://links.lww.com/AOG/C528).

We measured cytokines in milk from 26 of the 30 women who completed the questionnaire sections on side effects. Milk samples were selected to represent three time points: before vaccination, after the first vaccine dose, and after the second dose. For participants who reported side effects, the milk samples associated with the first day of reported side effects after each of the first and second doses were selected (Appendix 1, http://links.lww.com/AOG/C528). For participants who reported no side effects, the samples 1 day after the first and second doses were selected. Cytokines were measured in a multiplex assay (Mesoscale Discovery) according to the manufacturer’s instructions using 10-plex human V-PLEX Proinflammatory Panel 1 plates (K15049D). Each 96-well plate included an 8-point standard curve and assays for 10 cytokines: interleukin (IL)-2, IL-4, IL-6, IL-8, IL-10, IL-12p70, IL-13, IL-1β, interferon (IFN)-γ, and tumor necrosis factor-α. All samples and standards were run in technical duplicates.

Participant characteristics with continuous outcome measures are reported as mean and range, and characteristics with categorical outcomes are reported as percentages. The thresholds for positivity for RBD-specific antibodies were set at optical density values three times above the SD of the optical density values obtained with only the secondary antibody (background).^20,21^ For the neutralization assay, the concentration of antibodies that inhibited the binding of ACE2 to the spike or its variants (percent inhibition) was computed using the equation in Appendix 1 (http://links.lww.com/AOG/C528). Matched paired *t* tests were used to analyze differences between percent inhibition of neutralizing antibodies in milk provided before vaccination and milk provided after the second dose. Matched paired *t* tests were used to analyze differences in cytokine levels between the indicated timepoints. Independent *t* tests were used to analyze differences in infant stool anti-RBD antibodies, stratified by the presence of maternal side effects to immunization. Pearson R was used to determine the correlation between anti-RBD IgG levels and percent inhibition of SARS-CoV-2 antigens (neutralization). *P*<.05 was considered statistically significant. Statistical analyses were performed using GraphPad Prism 9.

## RESULTS

Thirty lactating women receiving mRNA-based COVID-19 vaccines were enrolled: 27 self-identified as White, one as Black, and two as Asian (Table 1). Overall, the number of women who reported experiencing any side effects was greater after they received the second dose as compared with after the first dose (Table 2).

**Table 1.**
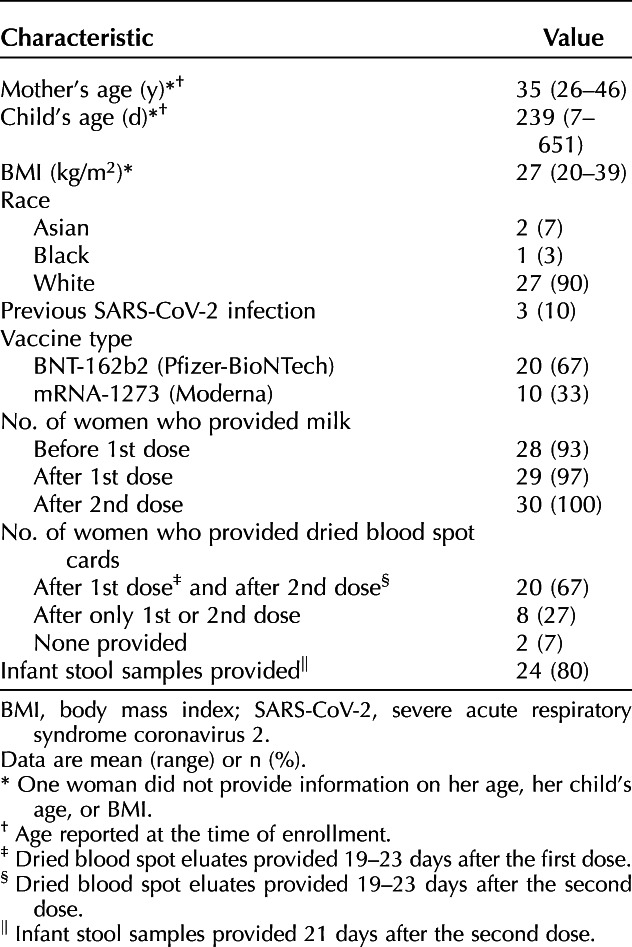
Participant Demographics and Vaccination-Related Information (N=30)

**Table 2.**
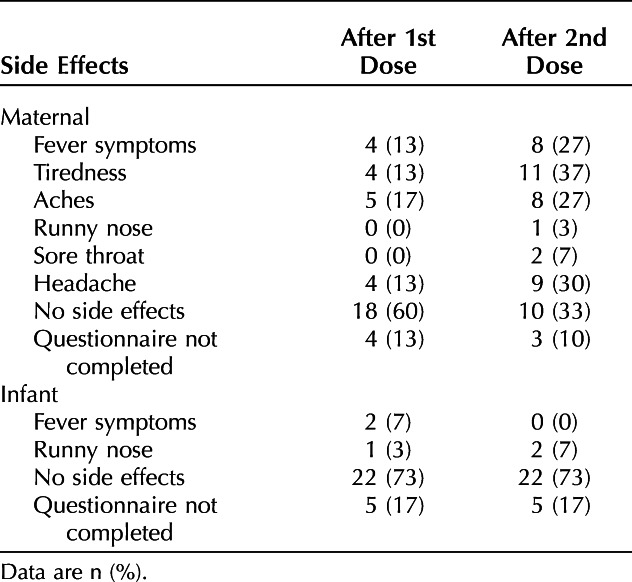
Maternal and Infant Side Effects

**Fig. 1.**
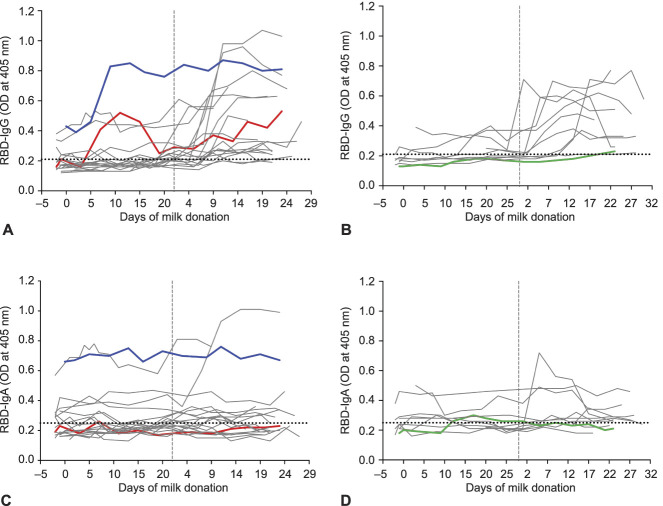
Antibody levels in serial milk samples after coronavirus disease 2019 (COVID-19) mRNA vaccination. Milk samples were obtained before the first dose (timepoint 0), across 19–23 days after the first dose, and across 19–23 days after the second dose (*vertical dashed line*) from 30 women vaccinated against severe acute respiratory syndrome coronavirus 2 (SARS-CoV-2). Whey fractions were assessed with enzyme-linked immunosorbent assay for receptor-binding domain (RBD)–specific immunoglobulin (Ig)G **(A, B)** and IgA **(C, D)**. *Colored lines* indicate serial milk samples obtained from three women who had a previous positive diagnosis of COVID-19. *Horizontal dotted lines* indicate the positive cutoff values. **A** and **C** are from women who received a Pfizer vaccine; **B** and **D** are from women who received a Moderna vaccine. OD, optical density. Narayanaswamy. COVID-19 mRNA Immunization in Lactating Women. Obstet Gynecol 2022.

**Fig. 2.**
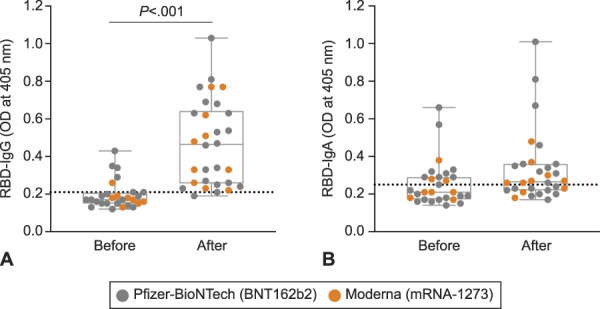
Immunoglobulin (Ig)G–dominant humoral response detected in the breast milk of women who received an mRNA-based coronavirus disease 2019 (COVID-19) vaccine. Comparison of levels of receptor-binding domain (RBD)–specific IgG **(A)** and IgA **(B)** in milk provided before vaccination and 3 weeks after the second dose. *Dotted horizontal lines* indicate positive cutoff values. OD, optical density. Narayanaswamy. COVID-19 mRNA Immunization in Lactating Women. Obstet Gynecol 2022.

We measured RBD-specific IgA, IgG, and IgM in serial milk samples of the 30 vaccinated participants. Overall, IgM levels were consistently negligible, and data are not shown. Milk samples provided more than 2 weeks after the second dose by 26 of the 30 women were positive for RBD-reactive IgG, albeit with a dynamic range (Fig. 1A and B). In contrast to IgG responses, milk samples from only 14 of 30 women were positive for RBD-reactive IgA (Fig. 1C and D and Fig. 2B). Lactational stage, as measured by the child’s age, was not related to the immune response in milk; milk from women with children aged 1.5 months and 23 months had comparable levels of anti-RBD IgA and IgG (Appendix 2A and B, available online at http://links.lww.com/AOG/C529).

Among the 30 vaccinated women, three reported that they had a prior positive test result for SARS-CoV-2. Two of the three women received the Pfizer vaccine (P1 and P2; Fig. 1A and C, blue and red lines), and one received the Moderna vaccine (P3; Fig. 1B and D, green line). Time from positive diagnosis to receiving the first vaccine dose was 210, 77, and 56 days for P1, P2, and P3, respectively. Of the three women, only P1 had a high level of anti-RBD IgA in milk obtained before vaccination (before day 0), and these levels remained consistent across all time points of milk donation (Fig. 1C, blue line). Unlike anti-RBD IgA, the level of anti-RBD IgG increased by approximately twofold 10 days after participant P1 received her first dose and then stayed high across later time points (Fig. 1A). Milk obtained before vaccination from participants P2 and P3 was negative for anti-RBD antibodies (Fig. 1A–D), despite positive SARS-CoV-2 test results 77 days (P2) and 56 days (P3) before initial vaccination.

Breast milk and serum humoral response to the vaccines were primarily IgG-driven. Eight of the 30 women provided dried blood spot cards at only one timepoint, and two women did not provide dried blood spots samples (Table 1). Serum RBD-specific IgA levels were at or below background in dried blood spots eluates from all 20 women (data not shown). Similarly, median RBD-specific IgA did not increase in milk from women after the second dose compared with prevaccine milk (Fig. 2B). In contrast, median RBD-specific IgG increased in milk from women after the second dose (Fig. 2A), along with an increase in median anti-RBD IgG in serum (Appendix 3, available online at http://links.lww.com/AOG/C529).

For comparison, we assessed RBD-specific antibodies in a set of 12 prepandemic milk samples (collected during January 2018–September 2019). Median levels of milk RBD-IgA and RBD-IgG (Appendix 4, available online at http://links.lww.com/AOG/C529) were below positive cutoff values established for vaccinated participants. However, three samples scored positive for anti-RBD IgA, and two samples scored positive for anti-RBD IgG.

**Fig. 3.**
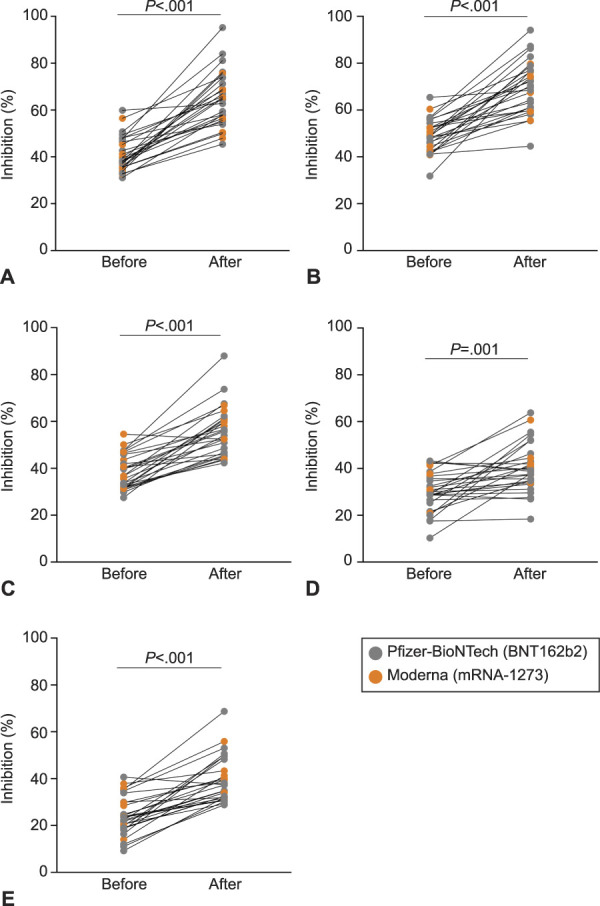
Milk samples from vaccinated women have neutralizing capacity against severe acute respiratory syndrome coronavirus 2 (SARS-CoV-2) spike complex. Comparison of percent inhibition of spike **(A)** and its variants of concern between milk provided before vaccination and 3 weeks after the second dose. Matched paired *t* tests were performed to analyze differences in percent inhibition before and after vaccination. D614G **(B)**, Alpha (B.1.1.7) **(C)**, Beta (B.1.351) **(D)**, and Gamma (P.1) **(E)**. Narayanaswamy. COVID-19 mRNA Immunization in Lactating Women. Obstet Gynecol 2022.

To determine functionality, we evaluated the presence of vaccine-elicited neutralizing antibodies in milk samples of the 28 women who provided prevaccination samples (Table 1). The milk samples from before vaccination and the milk samples that had the highest RBD antibody levels (Fig. 1 and Appendix 1 [Appendix 1, http://links.lww.com/AOG/C528]) were assayed for each woman. Milk neutralizing antibodies to spike and four variants of concern (D614G, Alpha, Beta, and Gamma) were evaluated.^22^ Antibody neutralization is reported as percent inhibition of binding by purified ACE2 to immobilized viral targets. These targets represent the viral spike complex of the original SARS-CoV-2 (spike) or those of mutated viruses that subsequently came to predominate at various times and locations (four characterized variants of concern). Analysis of paired samples shows that milk provided more than 3 weeks after the second dose was able to inhibit the binding of ACE2 to the spike (*P*<.001) (Fig. 3A) and to four variants of concern (Fig. 3B–E); however, inhibition for the Beta variant (B.1.351) was more limited (Fig. 3D). The neutralizing ability of milk weakly correlated with levels of RBD-specific IgG for spike and three of the four variants of concern (Appendix 5, available online at http://links.lww.com/AOG/C529 ). There was no relationship between lactation stage (child’s age) and milk anti-RBD IgA, anti-RBD IgG, or neutralizing capacity (Appendix 2, http://links.lww.com/AOG/C529).

**Fig. 4.**
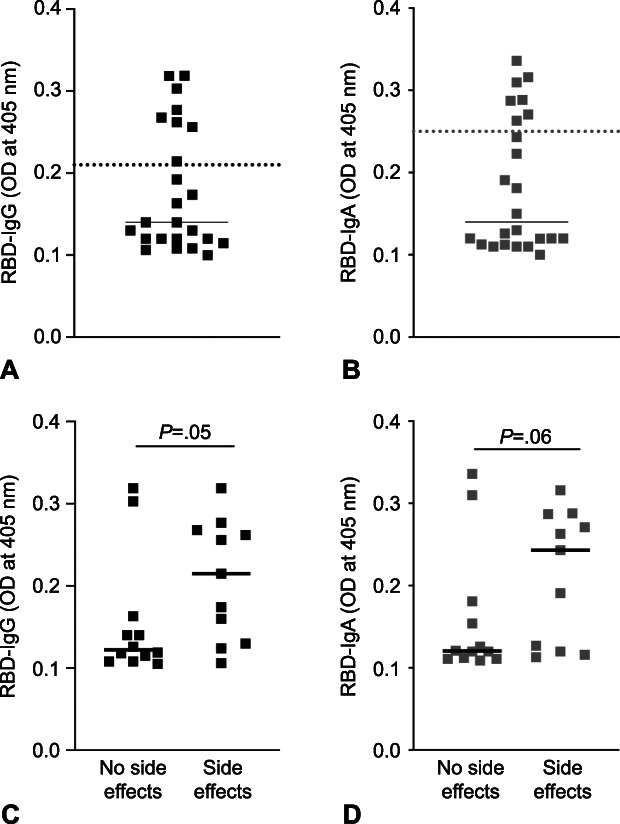
Anti–receptor-binding domain (RBD) immunoglobulin (Ig)G and IgA levels in infant stool are higher in infants of mothers who reported side effects after vaccination. Levels of anti-RBD IgG **(A)** and anti-RBD IgA **(B)** in stool samples obtained from infants (n=24) of vaccinated mothers. *Horizontal dotted lines* indicate positive cutoff values. Comparison of levels of anti-RBD IgG **(C)** and anti-RBD IgA **(D)** between stool obtained from infants of mothers who reported no side effects and those who reported vaccine-related side effects. Differences in levels of infant stool anti-RBD antibodies stratified by the presence of maternal side effects were assessed with independent t tests. OD, optical density. Narayanaswamy. COVID-19 mRNA Immunization in Lactating Women. Obstet Gynecol 2022.

We detected anti-RBD IgA and IgG in 30% and 33% of infant stool samples, respectively (Fig. 4B and A); these infants ranged in age from 55 days to 11 months. Total IgA and IgG in infant stool samples was measured as a validation of sample processing. Median total IgA and IgG levels were above positive cutoff limits (Appendix 6, available online at http://links.lww.com/AOG/C529). Prepandemic stool samples (n=6) contained IgA and IgG (Appendix 6, http://links.lww.com/AOG/C529) but were negative for anti-RBD antibodies (Appendix 4, http://links.lww.com/AOG/C529). We found that levels of anti-RBD IgG antibodies in infant stool were higher when immunized mothers had experienced side effects to the vaccine (Fig. 4C and D).

**Fig. 5.**
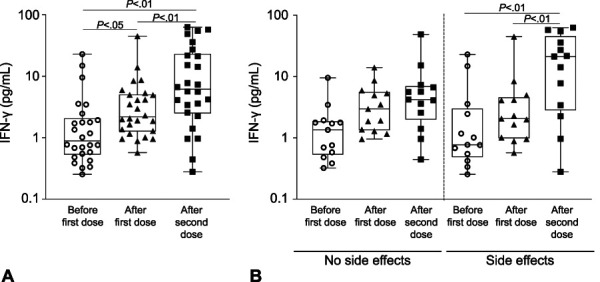
Interferon (IFN)-γ levels increase in milk after women are vaccinated. **A.** IFN-γ levels were assessed in milk obtained from women (n=26) at three timepoints: before coronavirus disease 2019 (COVID-19) vaccination, after the first dose, and after the second dose. **B.** IFN-γ levels assessed in milk at the three timepoints after stratifying women by whether they reported no side effects or side effects after either the first or second dose. *Horizontal lines* in each box indicate median concentration (pg/mL). Narayanaswamy. COVID-19 mRNA Immunization in Lactating Women. Obstet Gynecol 2022.

We measured levels of 10 key cytokines in milk of the 26 vaccinated lactating women who completed the questionnaire on side effects (Table 2). The levels of IFN-γ were significantly higher in milk provided after the first dose and after the second dose as compared with milk provided before receiving the vaccine (*P*<.05 and *P*<.01, respectively, Fig. 5A). For women who reported any side effects (n=13), compared with samples provided before vaccination, the median levels of IFN-γ increased by approximately 2.5-fold in samples provided after the first dose and by more than 20-fold in samples provided after the second dose. Overall, among the women who reported any side effects, the levels of IFN-γ were significantly higher in milk provided after the second dose compared with milk provided before receiving the vaccine (*P*<.01) (Fig. 5B). Among the women who reported no side effects after either the first or second dose (n=13), compared with samples provided before vaccination, the median levels of IFN-γ increased by approximately twofold in samples provided after the first dose and by threefold in samples provided after the second dose (Fig. 5B). Levels of five of the seven other tested cytokines were comparable across the three timepoints (Appendix 7, available online at http://links.lww.com/AOG/C529); levels of the remaining two cytokines (IL-12p70 and IL-4) were not consistently detectable (data not shown).

## DISCUSSION

We identified 10 published studies on the immune response to an mRNA-based COVID-19 vaccine in breast milk of lactating women.^9–18^ The number of lactating women included in eight of these studies ranged between five and 31; two remaining studies included 84 and 110 lactating women.^17,18^ All studies measured levels of SARS-CoV-2–specific IgA and IgG and, in some cases, SARS-CoV-2–specific IgM. However, only one study assessed neutralizing ability of antibodies in milk to the spike complex.^11^ Importantly, none of these studies measured cellular responses to the COVID-19 vaccine in milk or the presence of anti–SARS-CoV-2–specific antibodies in breastfed infants. Our study adds new information on the vaccine-induced immune response: we show neutralizing ability of antibodies in milk against spike and four variants of concern that is IgG-driven and high levels of IFN-γ in milk. Additionally, we show for the first time the presence of anti-RBD antibodies in the stool of breastfed infants.

Breast milk from vaccinated women contained elevated levels of antibodies against RBD. Induction of RBD-reactive IgG in milk occurred after the second dose in 87% of lactating vaccinated women, and we also detected RBD-reactive IgG in blood from all women assayed. In contrast to the IgG response in milk, RBD-reactive IgA was detected in only 47% of lactating vaccinated women, and we did not observe a concomitant increase in anti-RBD IgA in blood. The predominate immunoglobulin class in milk and other secretions is generally a secretory form of IgA, with production linked to mucosal surfaces. The lack of a robust anti-RBD IgA response in breast milk and blood of vaccinated women can be attributed to the intramuscular route of administration of the mRNA vaccines.^23^ In addition, class switching to IgG occurs very quickly after COVID-19 vaccination^24,25^ in the absence of prior exposure, which explains why we did not detect RBD-reactive IgA in most milk samples. In a disease state where SARS-CoV-2 is mucosally acquired, we and others find that the immune response mounted is primarily IgA.^19,21,26–28^ This is seen for one of the three vaccinated women in our study who also previously tested positive for SARS-CoV-2 infection. She had high levels of anti-RBD IgA in her milk prevaccination. We detected SARS-CoV-2–specific IgG and IgA in prepandemic milk provided by two and three women, respectively (Appendix 4, http://links.lww.com/AOG/C529). We^19^ and others^26,29,30^ have observed this in prepandemic milk and serum samples. A prior infection by other coronaviruses likely elicited an antibody response that cross-reacted with the RBD of SARS-CoV-2.

We assessed the ability of antibodies in milk to neutralize spike and four circulating variants of concerns. Milk provided after women received their second vaccine dose had significantly increased neutralizing ability as compared with their matched prevaccine milk samples. The neutralization ability of milk was significant against spike and all four variants of concern; however, we observed considerable variability among women. The milk of nine women showed no change in neutralization ability against the Beta (B.1.351) variant after the second dose, despite exhibiting neutralizing ability against spike (Appendix 8, available online at http://links.lww.com/AOG/C529). Studies of postvaccination serum also show reduced neutralizing antibody titers against the B.1.351 variant as compared with spike,^8^ and Collier et al^11^ report limited serum and milk antibody titers from vaccinated lactating and pregnant women against the B.1.351 variant. The individual differences among the milk of women to selectively neutralize specific variants warrants further study.

In most cases, the side effects of COVID-19 mRNA vaccines include fevers, headache, or myalgia. The extent to which these side effects correlate with the vaccine-induced cytokine response has received little attention. We explored this relationship and found elevation of the cytokine IFN-γ in breast milk. Most side effects can be attributed to the production of type I interferons, which play an important role in enhancing immune response in the early stages after vaccination. Type I interferons act in synergy with IFN-γ (a type II interferon), and both are key cytokines induced after an mRNA vaccination.^31^ Interferons induce activation of dendritic cells, enabling these cells to present the translated RBD to naïve CD4^+^ and CD8^+^ T cells, which are part of the adaptive immune system. This is crucial because activated CD4^+^ T cells stimulate B cells to produce RBD-specific antibodies. In addition, interferons promote the formation of long-lived memory CD4^+^ and CD8^+^ T cells.^32,33^ Thus, a key finding of our study is the elevation of IFN-γ in milk of women receiving an mRNA vaccine. This should protect nursing infants against several viral respiratory tract infections, including SARS-CoV-2.^34,35^

Infants of mothers previously unexposed to SARS-CoV-2 presumably have an underdeveloped immune response to the virus. We assessed stool samples from infants of the mothers immunized with a COVID-19 mRNA vaccine. Prior studies have demonstrated the ability to detect IgA and IgG in infant stool^36,37^; we detected total IgA in all infant stool samples and total IgG in all but one stool sample, consistent with prior studies.^36,38^ We assessed anti-RBD IgA and IgG in infant stool of vaccinated mothers. Importantly, we detected anti-RBD IgA and IgG in 30% and 33%, respectively, of infant stool samples. That only a subset of infant stool samples tested positive for anti-RBD IgA and IgG can be attributed to the degradation of antibodies within the infant’s gut. On these lines, low levels in a third of infant stool samples can also be due to transcytosis of anti-RBD antibodies from the infant intestine to circulation.^39,40^ Detection of anti-RBD antibodies in infant stool provides compelling evidence for the immunity conferred to infants from vaccinated mothers. It is well known that IgA mediates an early neutralizing response at mucosal surfaces^27,41^; therefore, detection of anti-RBD IgA in stool samples in our cohort suggests protection of these infants from a potential SARS-CoV-2 infection not only systemically (contributed by anti-RBD IgG), but also at mucosal sites mediated by anti-RBD IgA. The major limitation of our study is the small sample size, which did not allow us to explore the reasons for different immune responses among women.

Collectively, we have shown that antibodies in milk can neutralize the spike panel, and, importantly, we demonstrate for the first time the detection of anti-RBD antibodies in stool samples obtained from the infants of vaccinated mothers. The ability to detect anti-RBD antibodies in breastfed infants provides compelling evidence of antibodies transferred through breast milk and may be a motivation for women to continue breastfeeding after receiving a COVID-19 vaccine.

## Figures and Tables

**Figure:**
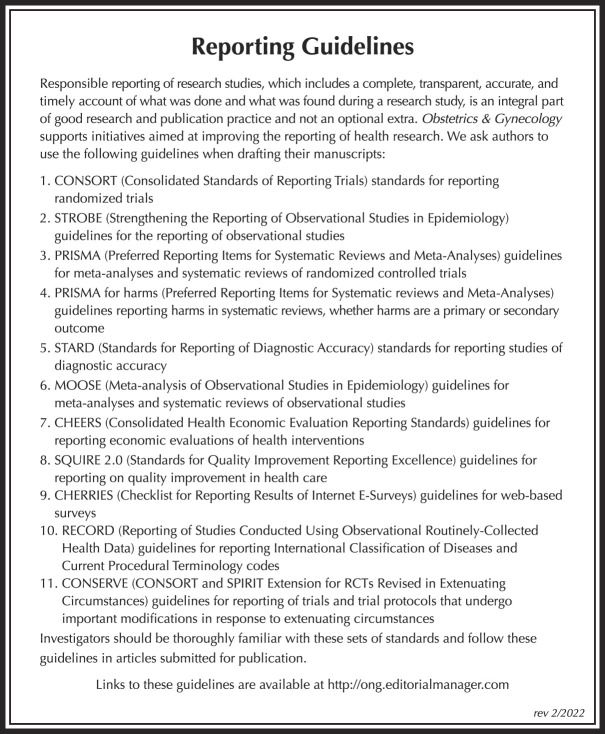
No available caption
